# The Ways of Forming and the Erosion/Decay/Aging of Bioapatites in the Context of the Reversibility of Apatites

**DOI:** 10.3390/ijms252011297

**Published:** 2024-10-21

**Authors:** Agnieszka Lasota, Mieczysław Gorzelak, Karolina Turżańska, Wojciech Kłapeć, Maciej Jarzębski, Tomasz Blicharski, Jarosław Pawlicz, Marek Wieruszewski, Mirosław Jabłoński, Andrzej Kuczumow

**Affiliations:** 1 Department of Maxillary Orthopaedics, Medical University of Lublin, 20-093 Lublin, Poland; agnieszka.lasota@umlub.pl; 2 Department of Orthopaedics and Rehabilitation, Medical University of Lublin, 20-059 Lublin, Poland; b.leszczynska@umlub.pl (M.G.); karolina.turzanska@umlub.pl (K.T.); wojciech.klapec@umlub.pl (W.K.); tomasz.blicharski@umlub.pl (T.B.); miroslaw.jablonski@umlub.pl (M.J.); 3 Department of Physics and Biophysics, Poznan University of Life Sciences, 60-637 Poznań, Poland; 4 Department of Orthopedics and Traumatology, Poznan University of Medical Sciences, 28 Czerwca 1956 135/147, 61-545 Poznań, Poland; jarek.pawlicz@gmail.com; 5 Department of Mechanical Wood Technology, Faculty of Forestry and Wood Technology, Poznan University of Life Sciences, 60-627 Poznań, Poland; marek.wieruszewski@up.poznan.pl; 6 ComerLab, Radawiec Duży 196, 21-030 Motycz, Poland; andrzej.kuczumow@gmail.com

**Keywords:** bioapatite, enamel and dentin formation, enamel erosion, enamel aging, bone formation, acid osteoporosis

## Abstract

This study primarily focused on the acid erosion of enamel and dentin. A detailed examination of the X-ray diffraction data proves that the products of the acid-caused decay of enamel belong to the family of isomorphic bioapatites, especially calcium-deficient hydroxyapatites. They are on a trajectory towards less and less crystallized substances. The increase in Bragg’s parameter d and the decrease in the energy necessary for the changes were coupled with variability in the pH. This was valid for the corrosive action of acid solutions with a pH greater than 3.5. When the processes of natural tooth aging were studied by X-ray diffraction, a clear similarity to the processes of the erosion of teeth was revealed. Scarce data on osteoporotic bones seemed to confirm the conclusions derived for teeth. The data concerning the bioapatite decays were confronted with the cycles of apatite synthesis/decay. The chemical studies, mainly concerning the Ca/P ratio in relation to the pH range of durability of popular compounds engaged in the synthesis/decay of apatites, suggested that the process of the formation of erosion under the influence of acids was much inverted in relation to the process of the formation of apatites, starting from brushite up to apatite, in an alkaline environment. Our simulations showed the shift between the family of bioapatites versus the family of apatites concerning the pH of the reaction environment. The detailed model stoichiometric equations associated with the particular stages of relevant processes were derived. The synthesis processes were alkalization reactions coupled with dehydration. The erosion processes were acid hydrolysis reactions. Formally, the alkalization of the environment during apatite synthesis is presented by introducing Ca(OH)_2_ to stoichiometric equations.

## 1. Introduction

Bioapatites are the basic materials for the formation of hard tissues among vertebrates. Bioapatite [1] is an inorganic calcium phosphate compound (CaP) in a special apatite crystallographic arrangement [2] and is of biological origin. It is not a pure apatite—it involves many minor and trace elements, primarily comprising magnesium, sodium, and carbonates, among others. Hard tissues include bones, skulls, and teeth. Hard organs are subject to continuous processes of maturation, aging, and erosion. All the erosion processes can be accelerated by mechanical and chemical influences, probably also by aging and material fatigue phenomena. The most noticeable are the processes of teeth erosion, which can be observed even with the naked eye on surfaces that are open to the environment. Better than observation by the eye, more efficient methods of physicochemical and spectroscopic characterization are used for the study of caries, as described by Yoshi et al. [3]; studies sometimes use multiple techniques [4] which complement one another. Unfortunately, the majority of those techniques are either of a qualitative character only or are not sufficiently quantified, especially for the observation of the dynamic processes of synthesis/decay. However, efforts are being made to understand the dynamical processes [5]. Such methods can be applied either in vitro or in vivo. All bioapatite decays are highly undesirable events since they lead to the lowering of life standards, severe suffering, and, in extreme cases to diseases, early retirement, constant disability, or even death from coupled diseases. This affects about 2.3 billion people worldwide [6,7]. It is estimated that, among people with deciduous teeth, the problem is serious for 30–50% of the population, while for people with permanent teeth, about 20–45% of them are affected, with a higher occurrence among males. This estimation is very rough due to the essential lack of data from many countries of the Global South. However, the statistics are not complete even in the most developed countries. The situation is even worse when regarded in the context of access to professional dental care. However, besides professional care, there are many available protective medicaments and procedures, which are most often helpful [8,9]; nevertheless, their effectiveness is sometimes doubtful [10]. The problem becomes a civilization disease, since our way of nutrition tends to involve more and more harmful components of a more acidic character which are poorer in calcium. The economic losses are counted in multi-thousand-dollar sums for individuals and multi-billion-dollar sums for whole societies; though these, as a rule, are divided into dental [11,12] and orthopaedical parts. Some now-somewhat-outdated numbers say that the costs of dental treatments were equal to 4.6% of total health treatments of any kind on a global scale [13]. Due to the great variability in caries, special criteria were elaborated for detailed diagnosis and further intervention in clinical practice [14,15,16,17,18,19,20]. Also, methods have been identified in anticipation of the future of tooth caries in endangered populations [21]. The problems of bone aging and osteoporosis are also devastating for society. This situation has for years motivated researchers to make intensive efforts to explain all the reasons for the degradation of bioapatites [22,23].

Undoubtedly, a complete understanding of the erosion process would be very helpful both for avoiding caries and—in cases where this is unsuccessful—in arresting or even inverting the defects. The main aims of this contribution are as follows: (i) to present the chemical and physical changes during progressing caries at the level of the main inorganic components of bioapatites in natural teeth and bones; (ii) to shift our analysis to a particle level; (iii) to evidence the crystallographic variability in bioapatites during the erosion process; (iv) to relate it to the processes of synthesis/decay of mineral apatites, aiming to identify the analogous intermediate phases, with full awareness of the deep differences; (v) to establish whether or not our ordering of bioapatites from [24] is correct or should be changed.

## 2. Results

### 2.1. Structural Changes in Up-to-Now Studies

Numerous papers have focused on the description of the natural, dietary [25], biological, mechanical [26,27,28], and chemical changes [29,30,31,32,33] in bioapatites. The acid-caused changes are probably the most severe and frequent among chemical changes due to the overconsumption of soft fruit juices [34,35], the excretion of acids on the surface of enamel plaque, and sometimes as a result of reflux disease [36]. However, even base-born erosion is sometimes considered [37]. The results are not always conclusive and comprehensive, and studies must be continued. One of the main problems relies on the fact that, although one can relatively easily determine the more-or-less-accurate initial chemical formula of the sound tissue, we have no further formulae expressing the next phases of decaying tissues during the selected moments. Also, the equations describing the progress of both hard tissue formation and hard tissue decay are essentially not very precisely elaborated. One of the reasons for this is the lack of general agreement concerning the stoichiometry of bioapatites. Below, some up-to-date proposals will be cited.

The chemical formulae for the reaction leading to the nonadvanced stages of erosion might be the ones proposed by Ingram and Silverstone [38], here somewhat modified:

Ca_10_(PO_4_)_6_(OH)_2_ + 2xH^+^ + 3H_2_O → Ca_10−x_(HPO_4_)_x_(PO_4_)_6−x_(OH)_2−x_∙(x + 3)H_2_O + xCa^2+^ (1)

with calcium-deficient hydroxyapatite (CDHA), Ca_9_(HPO_4_)(PO_4_)_5_OH∙4H_2_O, when we introduce x = 1 or octacalcium phosphate (OCP), Ca_8_(HPO_4_)_2_(PO_4_)_4_∙5H_2_O, as the substance resulting from the substitution of x = 2. This equation is important in the sense that the increasing value of the x coefficient expresses the progress in the decay reactions and it indicates the dynamical character of the reaction.

Next, we cite a similar but more conclusive equation, posited by Seredin et al. [39], because it indicates the products of decay; this is also more advanced than in Equation (1) (where An is an abbreviation of the anion):

Ca_10_(PO_4_)_6_(OH)_2_ + 8HAn + 10H_2_O → 6Ca(HPO_4_)∙2H_2_O + 4Ca(An)_2_ (2)

where brushite Ca(HPO_4_)∙2H_2_O is among the products. If one puts H_2_PO_4_^2−^ instead of An, the following equation will be valid:

Ca_10_(PO_4_)_6_(OH)_2_ + 8H_3_(PO)_4_ + 10H_2_O → 6Ca(HPO_4_)∙2H_2_O + 4Ca(H_2_PO_4_)_2_ (3)

**Figure 1:**
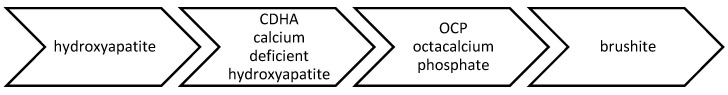
Structural changes from hydroxyapatite to brushite, resulting from stoichiometric equations shown in the text.

Please note that all the above reactions demand the action of acids and several products of decay are indicated, in the order shown in Figure 1.

An even more deepened and detailed equation by Featherstone and Lussi [23] is included here, where the enamel formula is closer to reality and the decay is going to the final stage of the total decomposition:

Ca_10−x_Na_x_(PO_4_)_6−y_(CO_3_)_z_(OH)_2−u_ F_u_ + 3H^+^ → (10 − x)Ca^2+^ + xNa^+^ + (6 − y)(HPO_4_^2−^) + z(HCO_3_^−^) + H_2_O + uF^−^ (4)

The above equations are ordered in such a way that they express more and more advanced erosion.

Now, we cite some experimental proofs from a great number of relevant papers. Arends and Davidson [40] and Glimcher et al. [41] claimed that brushite was evidenced in demineralized enamel. In another contribution, by Rowles and Levine [42], the octacalcium phosphate and optionally whitlockite (Ca_9_Mg(PO_4_)_6_(HPO_4_)) were found in the decaying dentine; in another by Featherstone et al. [43], the octacalcium phosphate and monetite Ca(HPO_4_) were detected in damaged enamel. Brown, Patel, and Chow [44] detected that brushite would arrive during enamel dissolution in much higher pH values than for the dissolving of pure hydroxyapatite. Lotsari et al. [45] and Habraken et al. [46] suggested a meaningful role of the complexes [Ca(HPO_4_)_3_]^4−^ and [Ca_2_(HPO_4_)_3_]^2−^, which could be related to the brushite. The comprehensive enlightening of the role of the brushite and the octacalcium phosphate in apatite synthesis is given in the paper by Johnsson and Nancollas [47]. The enamel should erode to the whitlockite and finally to brushite, according to suggestions by Rowles and Levine [42]. Similarly, in a deep study by Yoshihara et al. [48], the authors observed (Figure 3b in the paper by Yoshihara et al. [48]) the eroded structure with Ca/P = 1.25 and Ca/Mg = 6.75, which is relatively close to the whitlockite. In a previous study, we described the arrival of Mg in bioapatites, which entails strictly quantified amounts of water [49], favoring the reactions of decay. Altogether, it is—to some degree—the inversion of the synthesis methods; this explains the formation of bioapatites and finally hydroxyapatite, as presented in our paper [24]. We started from brushite and undertook a series of steps to obtain hydroxyapatite; this can be presented in a shortened form, at first via the octacalcium phosphate:

8Ca(HPO_4_) × 2H_2_O + 3H_2_O → Ca_8_(HPO_4_)_2_(PO_4_)_4_∙5H_2_O + 2H_3_PO_4_ (5)

and then

Ca_8_(HPO_4_)_2_(PO_4_)_4_∙5H_2_O + 2Ca(OH)_2_ → Ca_10_(PO_4_)_6_(OH)_2_ + 7H_2_O (6)

With the above equations side by side, one can obtain the following equation, which is very similar to inverted Equation (3):

8Ca(HPO_4_)∙2H_2_O + 2Ca(OH)_2_ → Ca_10_(PO_4_)_6_(OH)_2_ + 2H_3_PO_4_ + 4H_2_O (7)

It is worth noting that the key reagent in the reactions of synthesis was (OH)^-^ ion (sometimes formalized in the shape of Ca(OH)_2_). Otherwise, in the processes of decay (chemical erosion, Equation (4)), it is H^+^ (momentarily formalized as HAn). The process of decay is dynamic and irreversible in a natural way, although there are efforts to arrest or to reverse the course [50,51] and to rationalize the results from Equation (1), as compared with the equations from the paper by Kuczumow et al. [24]. The latter equations are written for the realistic averaged composition of the enamel. It does not change by anything in relation to Equation (4), except the volatilization of CO_2_ and the disappearance of defects, which introduces the elements of irreversibility. There is another interesting feature in Equations (1), (2), (5), (6) and (7). The ratio of Ca:P in brushite is 6:6; in octacalcium phosphate, it is 8:6; in whitlockite (not shown here) and idealized in amorphous calcium apatite, it is 9:6; and in hydroxyapatite, it is 10:6 [52]. Thus, in the processes of the synthesis of hydroxyapatite from the simpler compounds, continuous enrichment in calcium occurs. If we treated the erosion as the reversal of the synthesis, then one different process would occur, where the material is poorer and poorer in Ca in relation to P.

In some interesting trials of mathematical modeling, the formation of caries was also undertaken [53]. One of the most effective examples was that by Patel et al. [54], who calculated the mineral density profiles, formed under the influence of acetic buffer with pH = 4.5. The diffusion processes had to be taken into account, being dependent on the porosity. There have been numerous efforts to experimentally determine the composition of the decay products; it has been shown that it is possible for them to be identified as mineralogical entities [55], remains of enamel, or dentin; for example, this has been performed using Raman spectroscopy [56,57,58,59] and by other methods.

The main problem is that, in reality, we cannot determine many points of reference; the one that can be determined is the very first moment of the existence of unaffected hard bioapatite; such findings depend on the reasons, conditions, intensity, and timing of the erosion. The most-desirable modus operandi would be the conduction of systematic prolonged studies of the process with momentary measurements, registering consecutive results. The ideal model should begin with the mentioned starting point of the original intact enamel/bone; then, one would pass by the continuous registered changes up to the final point, i.e., the collapse of the crystallographic skeleton. The only promising method is to observe the continuous action of some damaging agent (e.g., not very strong acid [60]) and to detect the changes in one tooth. The experiment should be repeated to ensure statistical reliability, but then there is a problem with finding a possibly uniform tooth set. Another problem remains—can we have a method of detection that would allow us to register the changes without disrupting the experiment? Obviously, although dentists make contact with enamel and dentin erosions at different stages of the process, there are always different teeth, different patients, different erosion reasons, and different moments, which are impossible to rigorously coordinate.

### 2.2. Our Energy Changes Calculations

For the estimation of crystallographic changes, we use the calculations of energy changes associated with variations in crystallographic parameters d and sinΘ in the compound cell, as derived in our previous contribution [61]. They are as follows:

∆E = (6.2/d_1_)(1/sinΘ_1_ − 1/sinΘ_2_) (8)

∆E = (6.2/sinΘ_2_)(1/d_2_ − 1/d_1_) (9)

∆E = −6.2 × ∆d/(d^2^ × sinΘ) (10)

∆E = −6.2 × ∆sinΘ/(d_2_ × sin^2^Θ_2_) (11)

∆E = −(1/6.2) × ∆d × E^2^ × sinΘ (12)

And we should add one empirical equation:

∆E = k∆d(13)

where ∆E is the energy of the changes (essentially, the ion exchanges) and ∆d is the coupled change in Braggs’ crystallographic dimension (most frequently for the d(111) configuration). sinΘ is the sinus of constructive reflection angle, while d is generalized interplanar distance. As is shown, bioapatite isomorphic series is distinct from other isomorphic series of any compounds by its specific coefficient ‘k” in Equation (13).

It is obvious that we apply Braggs’ equation in an energetic manner, not using wavelength representation:

12.4n/E = 2dsinΘ(14)

while the value 1/d^2^ results to be in common hexagonal bioapatite:

1/d^2^ = 4/3[(h^2^ + hk + k^2^)/a^2^] + l^2^/c^2^ (15)

The mineral parts of bioapatite for enamel, apatite, dentin, and bone can be theoretically transformed by each other (together with all substituted cations and anions at their proper concentrations) and energies are calculated according to Equations (8)–(12). The results form a set of results obeying the dependence from Equation (13), and we consider them as a standard for isomorphic bioapatites.

### 2.3. Modelling of X-Ray Diffraction Results for Bioapatites

**Figure 2:**
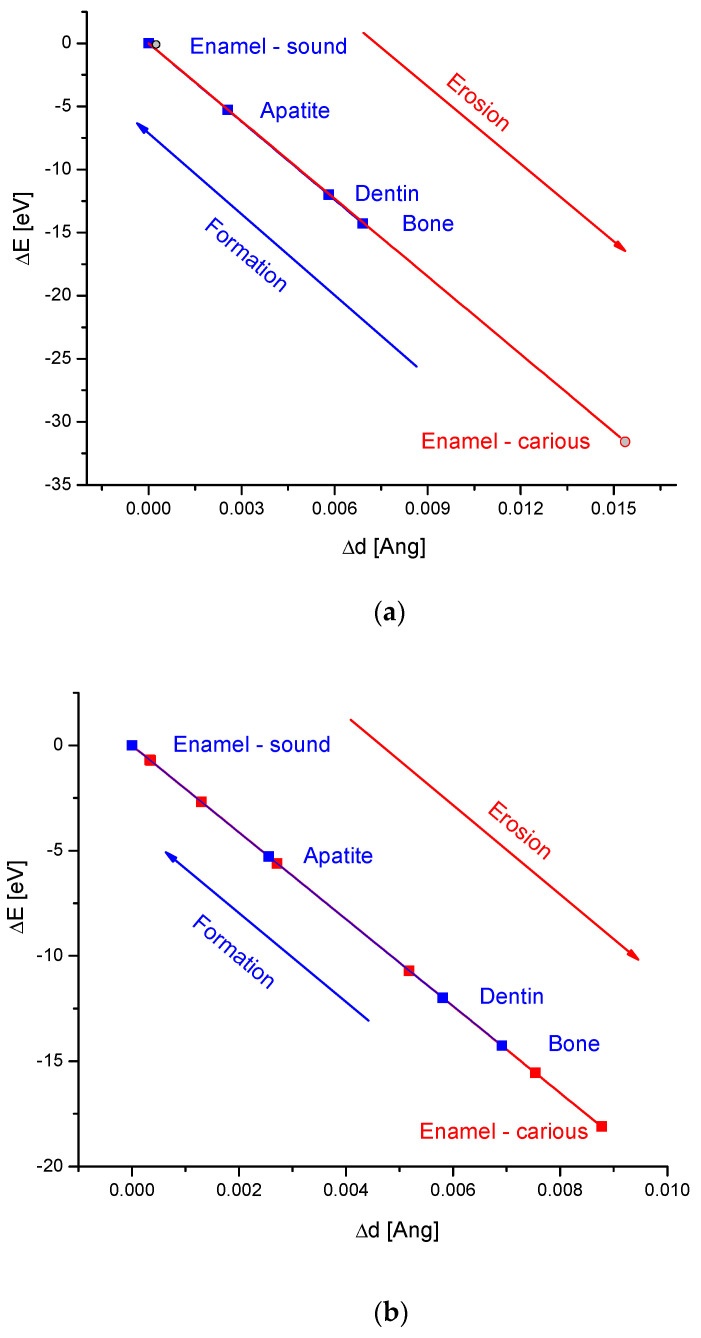
∆E-∆d diagrams for the placement of phases formed during the acid attack on the enamel, superimposed on the standard diagram for the basic bioapatites; (**a**) comparison of sound and carried locations from one patient [62]; (**b**) the same for the enamel treated with solutions of hydrogen peroxide with different pH levels [63]. The arrows show the directions of opposite reactions of formation (blue) and erosion (red). On both diagrams, the dental erosion drops below the level for the bone, the least-stable hard tissue.

There is not so much reliable data obtained from the uniform structural searches of the caries’ progress. The criteria are that we should find in the paper the X-ray diffraction data concerning the first point, i.e., the sound enamel, and strict data on the last point of the measurement (+potential intermediate points). The data can only concern the hexagonal phases due to the demands imposed by Equations (8)–(13). Alternatively, we should look for the monoclinic phases, but only the apatite optionally shows this arrangement. We selected only two relevant papers. The first of them presented the diffraction diagrams for the teeth of one child; the first diffraction diagram was characterized by the sound health of a tooth, and the other diagram showed a carious tooth (Katz et al. [62]) (Figure 2a). The second paper by Sabel et al. [63] concerned the action of hydrogen peroxide solution of different pH on enamel (Figure 2b). The data were superimposed on the standard ∆E-∆d curve made for the essential isomorphic bioapatites [64]. It is obvious that the decay of the enamel occurs along the same curve, which is characteristic of the isomorphous series of biological hard tissues. As one can see, the robust action of factors causing the erosion gives an overjump to the locations below the bone, the least advanced of the bioapatites. We must recognize that, in the system of bioapatite decay, we go beyond the scope of traditional human biomaterials—enamel, dentin, and bone. In the case of mild artificial acid enamel erosion at pH 4.5, we observe the energetical changes in the apatite, corresponding to the level of human dentin/bone (Figure 2b)—which is an insufficient level of mineralization, as for enamel. In the case of more drastic real erosion, the energetic level corresponds to the level far away outside. Still, the X-ray diffraction data indicate that newly erosion-formed compounds belong to the isomorphic family of bioapatites. Please note that the synthesis of consecutive bioapatites occurs along the same curve, but in a reverse direction (blue lines in the figures).

**Figure 3:**
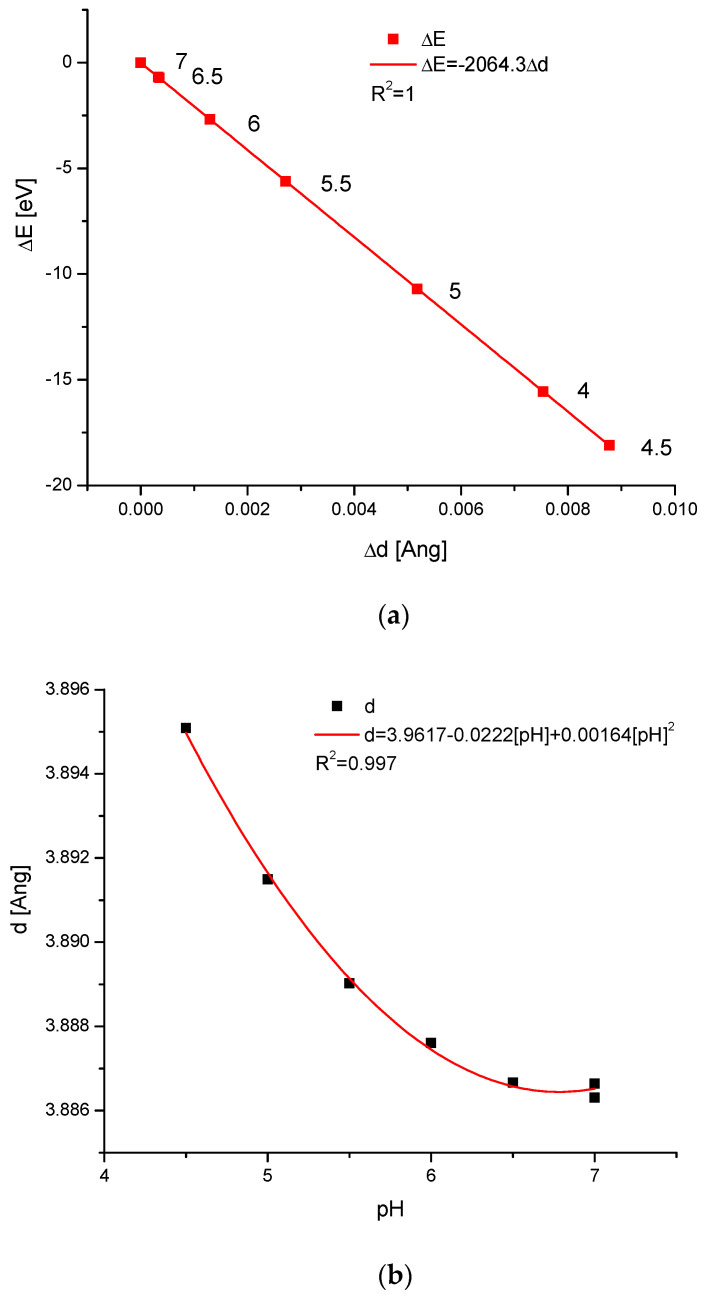

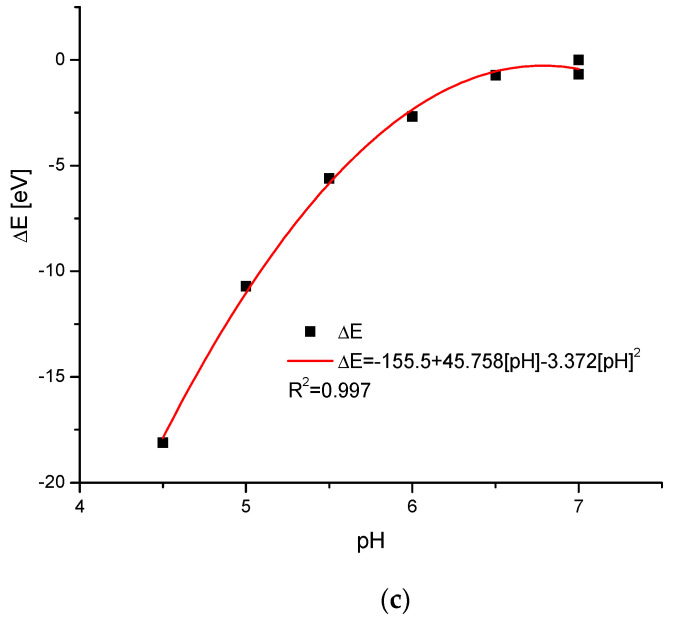
(**a**) Diagram ∆E-∆d for the enamel treated with the solutions of different pH (please note the slight disturbance at the level of pH 4); (**b**) changes in the d(1,1,1) parameter in Braggs’ equation and (**c**) in the energy of ion exchanges under the influence of changes in pH values (using data from the position in [63]). The erosion is presented from the right to the left side of (**b**,**c**), in opposition to (**a**). Please observe the stabilization areas close to neutral pH (**b**,**c**) and small energy corresponding with changes in such an area (**a**).

Figure 3 shows the changes in acidified enamel in more detail. It gives an image of how the changes in pH are mirrored in the ∆E-∆d diagram. Next, the action of pH on the value of the d parameter is presented (Figure 3b), and it is obvious that the acidification increases the dimension of the enamel apatite crystals. The energy of acid-modified crystals decreased. The pH values 6.5–7 belong to the stabilization range of enamel. Since the saliva pH is closed for normal circumstances in a range of 6.2–7.6, we suppose that it is not harmful to enamel. However, if the value drops below 5.5, which is observed during the drinking of soft juices, the real erosion starts. Here, we can observe the remineralizing role of saliva in pH close to the neutral one, while the demineralizing role is in a slightly more acidic range of pH [65,66].

**Figure 4:**
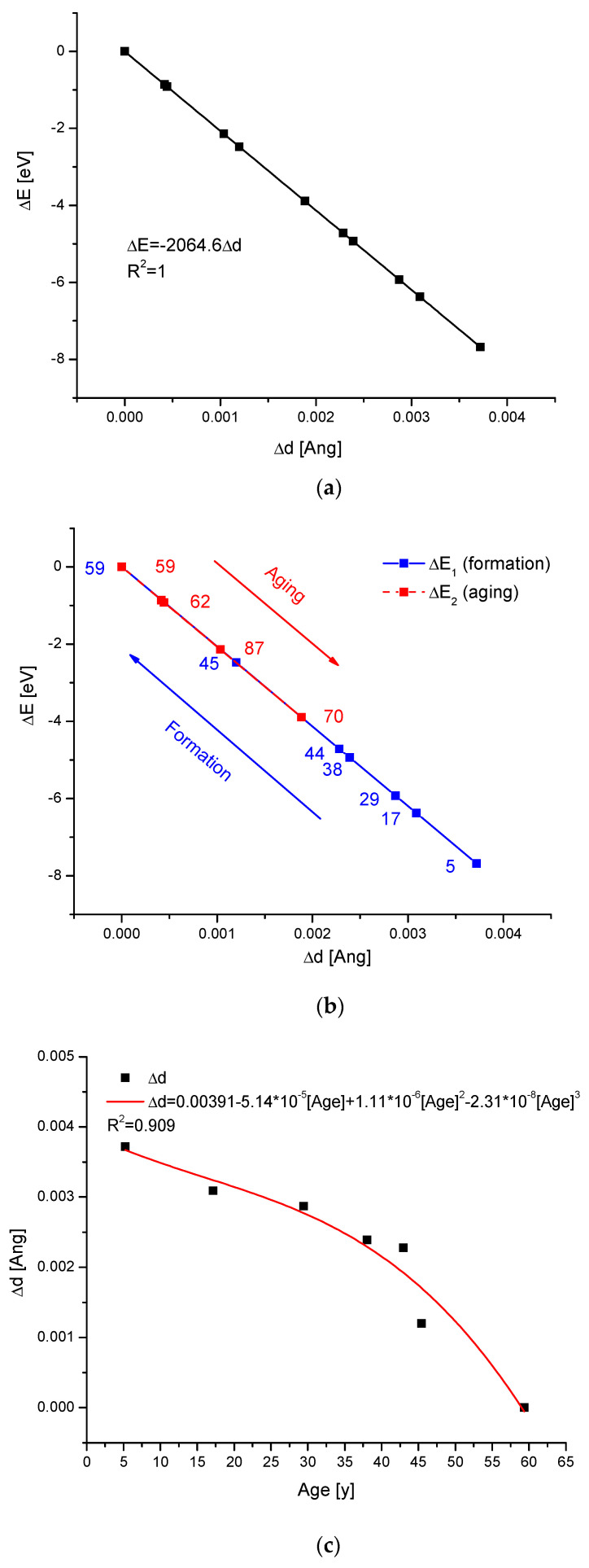

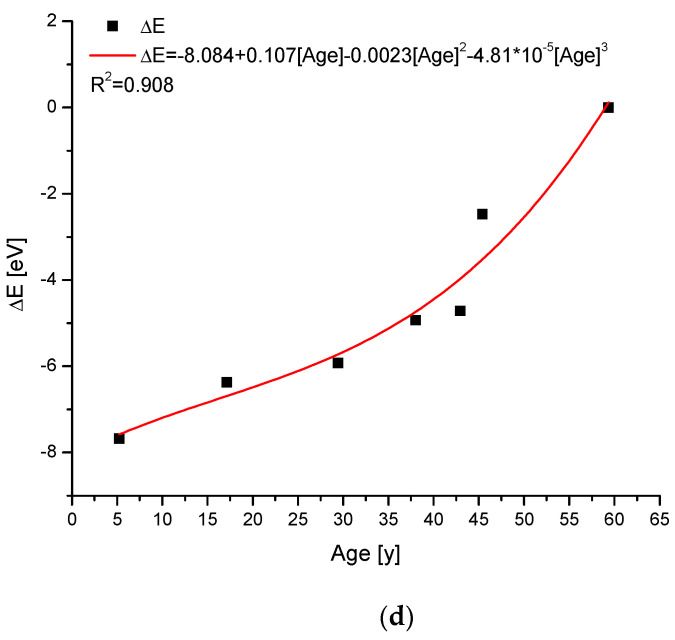
(**a**) The tooth samples related to age, as ordered in the diagram ∆E-∆d, were adopted from Leventouri et al. [67]. (**b**) The same results were divided on those concerning tooth maturation (blue) and tooth aging (red). (**c**) The change in the universal Braggs’ dimension d during the first phase of tooth aging. (**d**) The temporal variability in energy changes during tooth aging, at the first phase. The point (0,0) corresponds to the age of 59, which is the inversion point. The numbers associated with the points indicate the average age of patients. The reference levels for the figures were set on the data concerning the samples at age 59. (**c**,**d**) Quantitatively slow but constant processes of tissue changes.

Certainly, the fate of teeth is not always so dramatic, ending in total disruption. We know that the growth of human teeth is complex and that permanent teeth at first grow and become mature; next, at old age, they start to worsen (so-called aging); thus, we decided to also study this question. From Figure 4a, we can learn that all the results from older and older teeth lie on one straight ∆E-∆d curve. But the progress is reversible. When we decided to divide the points into those related to younger people and those related to older people, two kinds of curves were visible—the first one (red points) corresponded to the maturation of the teeth, and the second (blue points) corresponded to the aging process. There is a striking similarity between the sequence of processes maturation–aging, from Figure 4b, to the sequences of processes formation–erosion, from Figure 2. Processes connected with aging are clearly milder than those connected with erosion. The study of aging teeth concerns those studied by Leventouri et al. [67], which were rigorously documented at the age of 59. It is interesting that we can create separate curves, showing the energy change and universal dimension change vs. age, respectively. The curves under consideration (Figure 4c,d) are quite nice polynomials of the third order. They can both illustrate the physical details of the maturation process and be a basis for the estimation of tooth age.

Relatively, a very small amount of the relevant data concern the osteoporotic changes in crystallographic unit cell parameters. We managed to obtain single data by Sastry et al. [68], and the recalculation allowed us to make an estimate that the energy of the transformation of healthy bioapatite cells in osteoporotic one is <−25 eV for human femur heads. The drastic osteoporotic changes can be comparable with the values for eroded enamel.

### 2.4. Chemical Results for Apatites

**Figure 5:**
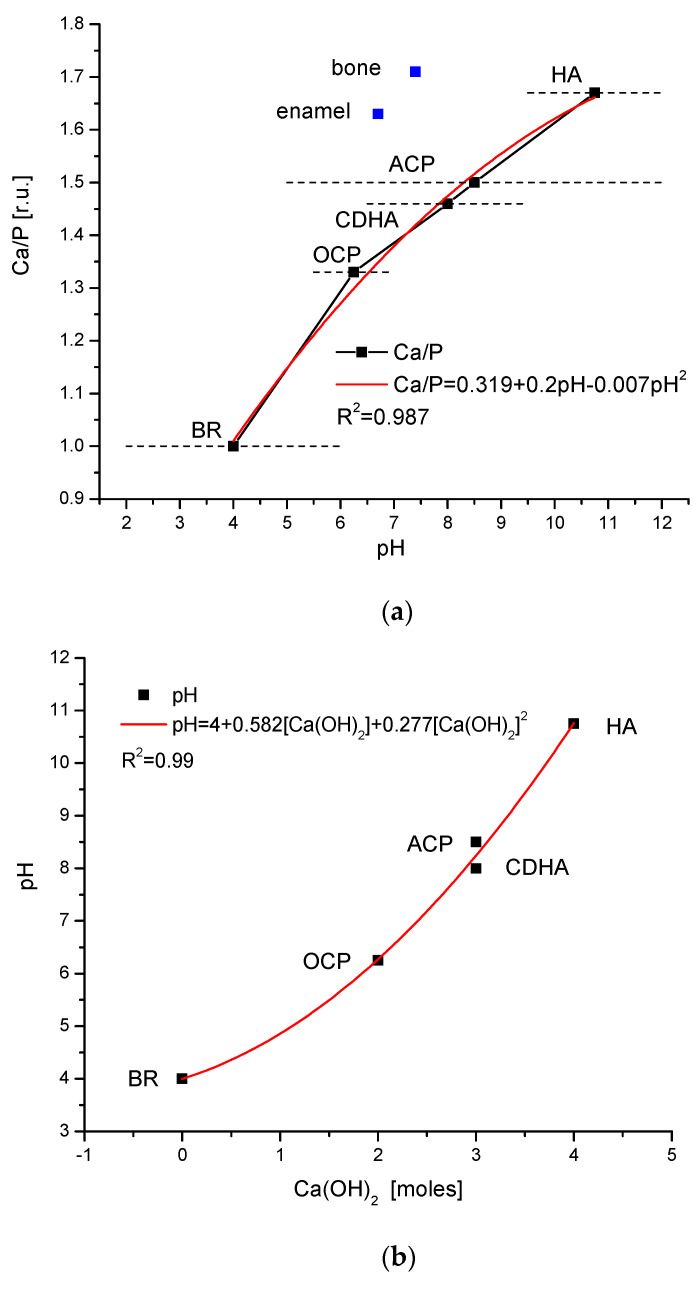

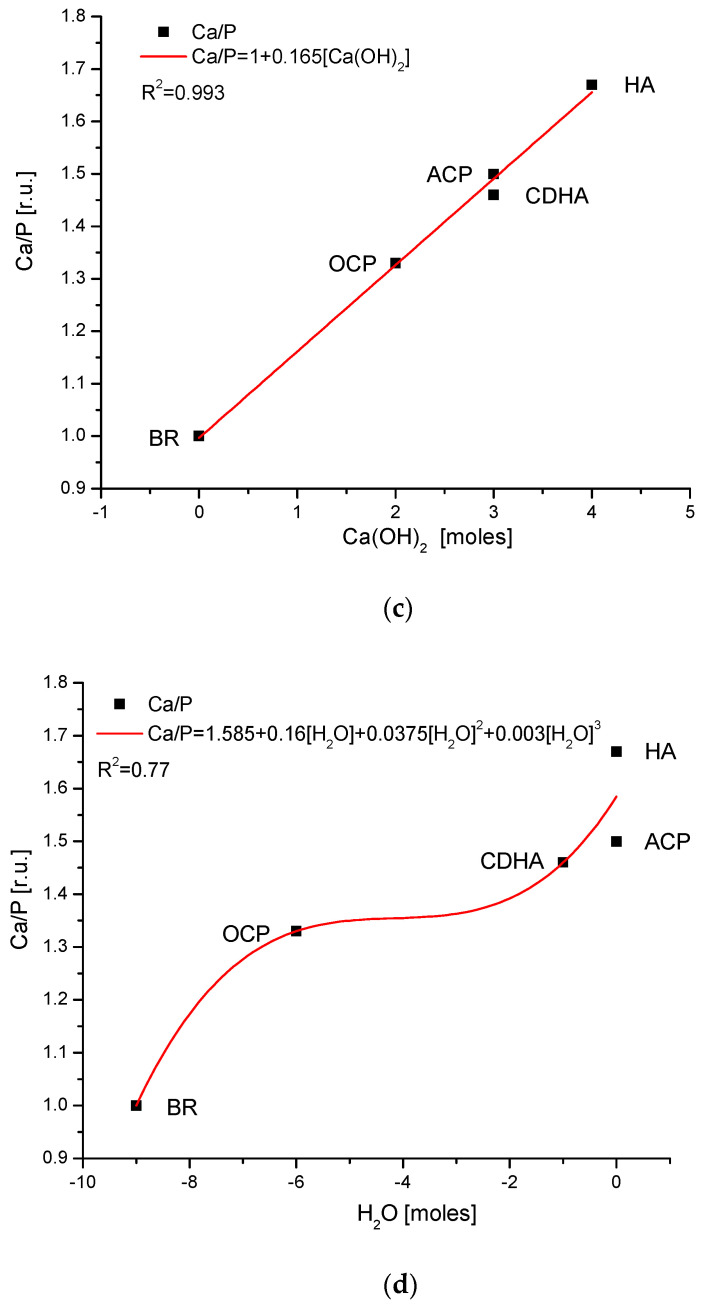
(**a**) The relationship between the Ca/P ratio and the pH range of stability for different precursor substances for the hydroxyapatite synthesis. The horizontal dropped lines show the relatively wide ranges of pH. As a basis for the construction of the diagram, the middle points of particular pH ranges were taken. See the great regularity of the curve. Areas of the real existence of bioapatites are shifted to minor values of pH (blue points). (**b**) Changes in pH values vs. amounts of Ca(OH)_2_ are necessary for the transformations of apatites according to Equations (1)–(4) in the text. (**c**) Variability in Ca/P ratio in relation to the added Ca(OH)_2_ corresponds to syntheses reactions in particular stages. See strictly linear dependence. (**d**) Hydrolysis of apatites, i.e., the inversion of synthesis.

The easiest way to explain the above data is through a consideration of the methods of pure apatite synthesis and determining whether the compounds mentioned in Section 2.1, as the precursors of the apatite, can be mutually ordered. The concept which seems to be related to the X-ray diffraction data considered earlier is the one which concerns the interrelation between the pH ranges of durability of particular phases from the one side and the Ca/P ratio of these compounds on the other side. The chemical phases are as follows: BR—brushite; OCP—octacalcium phosphate; CDHA—calcium-deficient hydroxyapatite; ACP—amorphous calcium phosphate; HA—hydroxyapatite; W—magnesium whitlockite. The pH ranges were taken from Dorozhkin [69] and Nikolenko et al. [70]. The relationship obeys a very simple, second-order polynomial relationship, with only a small second-order correction (Figure 5a). It is worth comparing with the somewhat different data by Larsen and Jensen [55], who claimed that brushite transforms into OCP at pH 7, while apatite transforms into OCP at pH levels above 8. On the other hand, the enamel apatite decays into brushite at pH 3.7, which is in accordance with what is observed in Figure 2 and Figure 3. According to Gray and Francis, the decay of brushite occurs in the pH range of 4.5–6 [71]. One must pay attention to the fact that the biomaterial was dissolved in their experiment and not the mineral apatite, which could be the reason for the deviation. We added the points concerning real enamel and bone to the figure. The relevant pH values were taken by considering that the durability of enamel must be optimized against the influence of saliva, the average pH of which is 6.7, while the durability of bone must be targeted in the pH of body fluids, which is 7.4 on average. It is clear that the conditions for the synthesis of bioapatites must be lowered by some pH units in comparison with the synthesis of mineral apatites. This is partially assured by the presence of Mg and carbonate ions, the lack of (OH)^−^ entities, and the presence of organic matter, forming a niche for the reactions. Nevertheless, please note that pH range for the bone and enamel durability is very close to the range for CDHA stability. It was only CDHA that arrived in the products of erosion in Figure 2, since we exclusively considered the results for the clearly observed hexagonal system. The order of compounds for the apatite cycle is strictly inverted versus the described processes of eroding of biomaterials.

In our consideration and equations explaining the results from Figure 5a, we claim that the consecutive attacks by Ca(OH)_2_ are decisive in further mineral transformations, in parallel with dehydration processes. It is interesting how this action translates to the values of the pH of the reaction. This is shown in Figure 5b. The relationship is perfect. But the dependence between the added Ca(OH)_2_ expressed in moles and the changed ratio if Ca/P is the most perfect. It is clear that those values are coupled in a linear manner. The reactions of dehydration are more complicated; nevertheless, they also may be profiled with quite a nice polynomial function (Figure 5d).

In the case of osteoporotic changes, up to the present, attention has been paid to the morphological variability, the estimation of mineral density, the dimension of crystallites, and the transformation of the organic environment rather than being paid to the detailed parameters of the apatite crystallographic cells. We can mention only the paper by Kourkoumelis et al. [72], where the authors estimated the decline of the Ca/P ratio in osteoporotic bones of rabbits against the same in the bones of healthy individuals. The drop was in the range of 9–10%, except for in the hips, where it was greater. Such a drop leads to values for the bone which are characteristic of ACP or CDHA—see the curve in Figure 5a.

## 3. Discussion

The results presented in Figure 2 are very important. We established earlier from the X-ray diffraction data that all the bioapatites together with the hydroxyapatite form a single series of isomorphic compounds [61]. In our recent contribution, the matter concerns erosion at first. The points of eroded enamel, for which it managed to measure the X-ray diffraction parameters, fall into one line, which is identical, but oppositely directed, to the line characteristic of the synthesis process. It testifies that the mineral erosion products formed under the acid influence in the range of pH 3/3.5–7 belong to the one unique family of isomorphic compounds; the basic part of the hexagonal structure is conserved. It can thus only concern calcium-deficient hydroxyapatites. The conclusion is that, during the erosion of bioapatite, the chemical processes occur in an inverted direction that is related to the processes that occur during the synthesis. It concerns acid-type erosion and has been established for the pH range up to ~3.5 in the lower range. In Equations (1)–(4), (5) and (6), a significant difference is observed: the reactions from the latter one (synthesis reactions) are occurring under the influence of OH^−^ (sometimes expressed in the shape of Ca(OH)_2_); meanwhile, in Equations (1)–(4) (decay reactions), it occurs under the influence of H^+^ cations in basic and acid environments, respectively. Equation (1) is fully compatible with the crystallographic results obtained by using the transformed Braggs’ law. Moreover, the data from Figure 2 testify that, during the enamel decay, the new phases are formed, less crystallized than the bone, but still being strictly isomorphic with all the structures involved in the standard diagram ∆E-∆d.

The data from Figure 3 and the observations by Seredin et al. [39] are in full accordance with the previous discovery by Driessens [73]—below pH 4.5, the hydroxyapatite becomes unstable and calcium-deficient; finally, brushite becomes the candidate for being the product of decay [74]. Lussi et al. [75] estimated that the critical value of the pH of plaque fluid is 5.5–5.7. However, our calculations show that this boundary can be shifted to the values of pH 6 (Figure 3b,c). The totally independent measurements confirmed that the products of the bioapatite decay belong to the family of isomorphic bioapatites, calcium-deficient hydroxyapatites (Figure 1), before they are transformed into other phases.

Figure 4 brings further confirmation of our reasoning. This time, the processes of tooth aging are considered. These results can be split into two parts—the first one illustrates the data for the patients under 59 years of age and the other one illustrates the data for older patients. The respective results for younger patients lay on one straight-line curve which increases. However, the results for older patients are also on a straight line, but which decreases. Moreover, both curves are the same lines, but they are in opposite directions. This is also the same curve as the standard curve diagram ∆E-∆d for the basic bioapatites. This testifies that the aging processes are opposite processes in relation to the formation of the tooth, and are somewhat similar to the above-mentioned processes of decay. The aging processes are much milder than the decay processes. There are announcements that bone aging is connected with delicate but continuous acidification of the blood environment [76].

The separate question is the location of values of the Ca/P parameter for the osteoporotic bones, which is known to be much lowered in comparison with the healthy bones. This demands some separate studies [72,77,78].

To explain the crystallographic and chemical results, we should write correct equations. For the results from Figure 5, the postulated equations are as follows:

6Ca(HPO_4_)∙2H_2_O (BR) + 2Ca(OH)_2_ ⇔ Ca_8_(HPO_4_)_2_(PO_4_)_4_∙5H_2_O (OCP) + 9H_2_O (16)

Ca_8_(HPO_4_)_2_(PO_4_)_4_∙5H_2_O + Ca(OH)_2_ ⇔ Ca_9_(HPO_4_)(PO_4_)_5_OH (CDHA) + 6H_2_O (17)

Ca_9_(HPO_4_)(PO_4_)_5_OH ⇔ Ca_9_(PO_4_)_6_ (ACP) + H_2_O (18)

Ca_9_(PO_4_)_6_ + Ca(OH)_2_ ⇔ Ca_10_(PO_4_)_6_(OH)_2_
(HA) (19)

Perhaps, the further action of Ca(OH)_2_ can deepen the reaction:

Ca_10_(PO_4_)_6_(OH)_2_
(HA) + 2Ca(OH)_2_ ⇔ Ca_4_(PO_4_)_2_O (TTCP) + Ca(HPO_4_)∙2H_2_O (BR) (20)

The yellow highlight emphasizes the action of an alkaline substance, the blue highlight indicates the presence of water, and the designations of the key substances are indicated with a green highlight. The ordering of equations strictly corresponds to the situation shown in Figure 5a. This set of equations emphasizes the chemical character of changes. From left to the right, the equations present the alkalization reactions with the dehydration, except the third equation, which concerns only the dehydration reaction. The alkalization is strong and demands four moles of Ca(OH)_2_ per one mole of hydroxyapatite. The participation of Ca(OH)_2_ in reactions was observed years before by Meyer and Eanes [79]. If the reactions are reverted, then they would concern the hydrolysis processes, possibly acid hydrolysis (16 particles of H_2_O). The above set of equations is very elegant, with constantly and logically changing chemical formulae of OCP, CDHA, and HA. One cannot consider that Ca(OH)_2_ exists as such in reactions—it is sufficient if, in some close locations, the respective ions arrive in the proper proportions and the total effect is as if it reacts Ca(OH)_2_. It is in part similar to discoveries made by Querido et al. [80]. One can treat OCP, CDHA, and ACP as autocatalysts; they arrive and then disappear in the reactions. Rather, we do not observe them among the primary reactants nor among the final products. Please note that the reverted equations from Equations (17)–(19) correspond to Equation 1 by Ingram and Silverstone [38], while Equations (16)–(19) correspond to Equation (5) and (6) by Seredin [39].

The situation is somewhat less logical from the crystallographic point of view. OCP shows a triclinic arrangement; then, it should transform in CDHA with a hexagonal structure [81,82]; then, this occurs in the ACP of an amorphous shape; then, once again, it occurs in the hexagonal HA. It indicates the continuous change in the crystallographic structure. To solve the problem, we propose an alternate set, Equations (21)–(24):

6Ca(HPO_4_)∙2H_2_O (BR) + 2Ca(OH)_2_ ⇔ Ca_8_(HPO_4_)_2_(PO_4_)_4_∙5H_2_O (OCP) + 9H_2_O (21)

Ca_8_(HPO_4_)_2_(PO_4_)_4_∙5H_2_O + Ca(OH)_2_ ⇔ Ca_9_(PO_4_)_6_ (ACP) + 7H_2_O (22)

Ca_9_(PO_4_)_6_ + H_2_O ⇔ Ca_9_(HPO_4_)(PO_4_)_5_OH (CDHA) (23)

Ca_9_(HPO_4_)(PO_4_)_5_OH + Ca(OH)_2_ ⇔ Ca_10_(PO_4_)_6_(OH)_2_ (HA) + H_2_O (24)

The sequence and kind of equations were changed. In the second equation, OCP is only mildly alkalized to ACP. In this set, we have the ACP → CDHA sequence, while in Equations (16)–(20), this occurs in an inverse order. Please observe that, in Figure 5a, the difference between those two substances is not great. In Equations (21)–(24), we have the same level of alkalization, which demands four moles of Ca(OH)_2_ per one mole of hydroxyapatite, and the same degree of dehydration (16 particles of H_2_O). Equations (18) and (23) in each set are inverted side by side: in Equations (16)–(20), it is dehydration; in Equations (21)–(24), it is hydrolysis. The above reactions probably participate in forming different bioapatites during some phases of the syntheses with the presence of organic matter, consecutively creating dentin, cementum, bone, and enamel, but in a much milder chemical environment. In an inverted direction, in the cases of erosion, CDHA, ACP, OCP, and even brushite can arrive as these products. We purposely neglected the presence of Mg, Na, and carbonates as well as the real, non-integer coefficient to emphasize the essential reactions.

Since the whitlockite is considered to come second in terms of the amounts of hard components in the bone [83], we can suggest some special set of reactions; these are denoted below:

6Ca(HPO_4_)∙2H_2_O (BR) + 2Ca(OH)_2_ ⇔ Ca_8_(HPO_4_)_2_(PO_4_)_4_∙5H_2_O (OCP) + 9H_2_O (25)

Ca_8_(HPO_4_)_2_(PO_4_)_4_∙5H_2_O + Ca(OH)_2_ ⇔ Ca_9_(PO_4_)_6_ (ACP) + 7H_2_O (26)

Ca_9_(PO_4_)_6_ (ACP) + Mg(HPO_4_) ⇔ Ca_9_Mg(PO_4_)_6_(HPO_4_) (WH) (27)

Ca_9_Mg(PO_4_)_6_(HPO_4_) (WH) + Ca(OH)_2_ ⇔ Ca_10_(PO_4_)_6_(OH)_2_ (HA) + Mg(HPO_4_) (28)

In comparison with Equations (16)–(24), we have the same level of alkalization and hydration. If the last reaction is stopped in between, then the mixture of whitlockite and apatite can be observed, as is claimed for the bones.

If one separately summarizes the sets of Equations (16)–(28) side by side, the obtained result is as follows for each time:

6Ca(HPO_4_)∙2H_2_O (BR) + 4Ca(OH)_2_ ⇔ Ca_10_(PO_4_)_6_(OH)_2_ (HA) + 16H_2_O (29)

which can be executed in separate stages; these confirmed that CDHA, ACP, and OCP are substances of the autocatalytic character.

The arrows on both sides were introduced to the equations in an intentional way. The equations, if read from the left to the right and one after another, express the formation of compounds towards the most advanced substance—hydroxyapatite. In the other direction, we have the decay/aging process. As we see from Figure 2, Figure 3 and Figure 4, the reactions of bioapatites are reversible in the crystallographic sense; from Figure 5, we can see that the reactions of apatites also occur in the chemical sense.

Such opinions about reversibility in the system of apatites and bioapatites have been expressed in the literature [84]. The role of acid-based parameters in such cycles was observed [85]. The dependence between Ca/P and pH was suggested by Layrolle and Daculsi [86]. Through using apatite-composed toothpaste, one can obtain positive remineralization of the enamel [87]; in other words, the inversion of the erosion occurs. One thing is striking—the huge difference in pH values for the reactions in apatite and bioapatite systems. Please remember that the stability range of enamel covers pH values 6.5–7.8 (which should be resistant against saliva); for bone, this range is pH 7.35–7.45 (the stability against body fluids); the HPO_4_^2−^/H_2_PO_4_^−^ buffer system works at pH values of 5.5–8; the interior of mitochondrial cells is sometimes alkalized up to pH 8.2 [88,89,90]. Thus, the widely understood environment for living cells/biomaterials covers the pH range 6.5–8.2 (although, see the paper by Lyman and Waddell, where the upper limit is pH 8.5 [91]). We suppose that the association of Mg and carbonates involved in bioapatites and the syntheses involved in specific organic matrices allow one to perform the reactions in much lower values of pH than those from the curve shown in Figure 5a, without the violation of the essential indications resulting from this figure. On the other side, the removal of carbonates during the erosion process brings an element of irreversibility. Please note that the areas of pH stability of CDHA cover a whole range of pH values that are allowed in human organisms (Figure 5a), while ranges of ACP and OCP touch that region.

The data concerning tooth aging allude to this essential breakthrough that occurs when a person reaches 59 years of age. This finding astonishingly coincides with a recently announced second breakthrough in human life [92], based on multi-omics profiles. Their results are centered around the age of 60 years. These theories demand further X-ray diffraction studies, especially on a set of sound human bones of uniform type, which should be collected from people who have been subject to accidents or from people who have died due to causes unrelated to bone disease. This direction of investigations was outlined by Leventouri et al. [67]. Such research would potentially elucidate the differentiation between changes in bones that result simply from aging and those induced by osteoporosis.

We disposed of only some very scarce data concerning the behavior of the single apatite crystallographic cells during the osteoporosis process. A great number of factors that can be associated with osteoporosis are described in detail in the literature; but, surprisingly, the chemical changes in single apatite cells are neglected in the literature. From studies on human cells, the change in the energy calculated by us reached −26.5 eV [68]. It is, in parallel, in accordance with the processes of erosion of teeth or the processes of aging. For the processes of osteoporosis of rabbit bones [72], the Ca/P parameter was lowered by ~10%, which corresponds to 0.17, measured in relative units; this is also in accordance with the process observed in human cases. If we compare the former data with the relationship derived from Figure 3c (if the dependence is more universal than it is for the case described in the figure), it corresponds to the lowering of the pH by 3 units. For the latter data and by referring to Figure 5a, we see a lowering by 2 units of pH. In general, in the mentioned cases, we can observe processes that are contractually equivalent to acid hydrolysis. Those results demand further detailed studies.

Although changes shown in Equations (16)–(29) and illustrated in Figure 5 concern mineral components, while those shown in Figure 2, Figure 3 and Figure 4 show biominerals and their resulting minerals, both systems are compatible—although they are sensitive to changes in pH (Figure 5a). It is important to note that we quantitatively established that the reactions of biomineral syntheses belong to a category of alkalization and dehydration reactions, while erosion/aging reactions are the acid hydrolyses reactions. Changes in pH or wetting conditions clearly influence the outcomes of these processes.

## 4. Materials and Methods

### 4.1. Basic Data

For our modeling process, the data were taken from previously published articles from reliable materials available in the literature, after very careful scrutiny. The sources are cited in the relevant fragments of the text.

### 4.2. Software

The calculations were conducted with the use of the Origin9.1 program.

### 4.3. Calculations

All the proposed stoichiometric relationships are written in the shape of equations with the integer coefficient. This was performed with full awareness that it corresponded only to the situations treated either in an idealized manner or in a partially ideal manner, in the mineralogical sense. Nevertheless, the arrays of bioapatites and apatites asymptotically reach the idealized formulae; this allows us to consider their transformations in a much easier and illustrative manner. In the same way, having the awareness of the differences between bioapatites and apatites, the latter in mineralogical and chemical senses, not identical, we try to exploit the deep similarities of the crystallographic shapes and chemical reactions.

## 5. Conclusions

A consideration of the sparse valuable data from systematic X-ray diffraction studies on the consecutive phases that occur during the continuous artificial erosion processes brings some very interesting results. We have established that the acid erosion processes of enamel can be rationalized by X-ray diffraction measurements. ∆E-∆d diagrams allowed us to determine that the action of acids in a range pH 3.5–7 gave erosion products which reflected a prolonged version of the standard scale for typical bioapatites. Moreover, the erosion in enamel occurs along the same crystallographic trajectory as the enamel-formation process, but in an opposite direction. Although the first points of such curves were typical for enamel, the last points, corresponding to the products of advanced acid erosion, jumped outside the calibration curve for the bioapatites, to the values well below the data for the bone material. Otherwise, it corresponded to the different stages of calcium-deficient hydroxyapatite, CDHA. The processes of tooth aging were considered as the next ones showed a striking similarity to very mild acid-induced tooth erosion. We can compare the similarities between the two reversible series: enamel formation–decay and enamel maturation–aging. On the basis of even more rare data, we suppose that the same relationship can be detected for bone synthesis and acid-type osteoporosis.

Since it is necessary to establish the order of compounds arriving during the decay process, the relationship between the Ca/P ratio and the range of pH stability for these compounds was studied; it turned out to be some regular function. This allowed us to derive the sets of equations describing the processes of synthesis/decay. The syntheses of apatites and bioapatites were the alkalization reactions joined with dehydration. The reaction of decay was of an acid hydrolytic character. The ranges of pH were different, but the decay of bioapatites and the synthesis of apatites reached the same range in the field of calcium-deficient hydroxyapatites.

## Data Availability

All data were taken from previously published papers. In the manuscript text, appropriate references were added.
